# Repurposing the oncolytic virus VSV∆51M as a COVID-19 vaccine

**DOI:** 10.3389/fbioe.2023.1150892

**Published:** 2023-07-17

**Authors:** Almohanad A. Alkayyal, Manar Darwish, Reham Ajina, Saleh Y. Alabbas, Mohammed A. Alotaibi, Abeer Alsofyani, Maha Bokhamseen, Maumonah Hakami, Omar A. Albaradie, Abdulaziz M. Moglan, Sharif Hala, Abdullah Faisal Alsahafi, Samer Zakri, Adnan Almuzaini, Khamis Alsharari, Feras Kaboha, Mustafa Y. Taher, Haggag S. Zein, Fayhan Alroqi, Ahmad Bakur Mahmoud

**Affiliations:** ^1^ Department of Medical Laboratory Technology, Faculty of Applied Medical Sciences, University of Tabuk, Tabuk, Saudi Arabia; ^2^ Immunology Research Program, King Abdullah International Medical Research Center, Riyadh, Saudi Arabia; ^3^ Department of Clinical Laboratory Sciences, College of Applied Medical Sciences, King Saud Bin Abdulaziz University for Health Sciences, Riyadh, Saudi Arabia; ^4^ Department of Cellular Therapy and Cancer Research, King Abdullah International Medical Research Center, Jeddah, Saudi Arabia; ^5^ King Saud Bin Abdulaziz University for Health Sciences, Ministry of National Guard Health Affairs, Jeddah, Saudi Arabia; ^6^ College of Medicine, King Saud Bin Abdulaziz University for Health Sciences, Jeddah, Saudi Arabia; ^7^ Infectious Disease Research Department, King Abdullah International Medical Research Centre, Ministry of National Guard Health Affairs, Jeddah, Saudi Arabia; ^8^ Experimental Medicine Department, King Abdullah International Medical Research Centre, Jeddah, Saudi Arabia; ^9^ College of Applied Medical Sciences, Taibah University, Madinah, Saudi Arabia; ^10^ Department of Microbiology and Immunology, Dalhousie University, Halifax, NS, Canada; ^11^ Department of Immunology, Ministry of the National Guard—Health Affairs, Riyadh, Saudi Arabia; ^12^ King Saud Bin Abdulaziz University for Health Sciences, Riyadh, Saudi Arabia; ^13^ Strategic Research and Innovation Laboratories, Taibah University, Madinah, Saudi Arabia; ^14^ Immunology Research Program, King Abdullah International Medical Research Center, Jeddah, Saudi Arabia

**Keywords:** COVID-19 pandemic, oncolytic virus, repurposing oncolytic virus, VSV∆51M-RBD vaccine, SARS-CoV2 vaccine

## Abstract

The coronavirus disease 2019 (COVID-19) pandemic imposes an urgent and continued need for the development of safe and cost-effective vaccines to induce preventive responses for limiting major outbreaks around the world. To combat severe acute respiratory syndrome coronavirus 2 (SARS-CoV-2), we repurposed the VSV∆51M oncolytic virus platform to express the spike receptor-binding domain (RBD) antigen. In this study, we report the development and characterization of the VSV∆51M-RBD vaccine. Our findings demonstrate successful expression of the RBD gene by the VSV∆51M-RBD virus, inducing anti-RBD responses without attenuating the virus. Moreover, the VSV∆51M-RBD vaccine exhibited safety, immunogenicity, and the potential to serve as a safe and effective alternative or complementary platform to current COVID-19 vaccines.

## Introduction

Severe acute respiratory syndrome coronavirus 2 (SARS-CoV-2) emerged in Wuhan, the capital of Hubei Province in China, and rapidly caused a pandemic in 2020. Despite the efforts made to contain the spread of this virus, as of 20 January 2023, there have been over 663 million confirmed cases and over 6.7 million deaths reported globally (WHO, 2023). The urgent need for an effective vaccine led to an unprecedented effort to develop and test multiple vaccine candidates. Currently, there are at least 21 COVID-19 vaccines approved globally for emergency use (Rahman et al., 2022). These vaccines have been developed using different platforms, including inactivated, live attenuated, protein subunit, virus-like particle (VLP), viral vector, DNA, and RNA platforms. Although they have some similarities in activating immune responses against SARS-CoV-2, they also possess some differences in their immunogenicity and efficacy profiles (Frederiksen et al., 2020; Rahman et al., 2022).

SARS-CoV-2 is classified into the Coronaviridae family, which is characterized by an enveloped structure with a positive-sense single-stranded RNA (+ssRNA) genome. The viral genome encodes four structural proteins, known as the spike (S), envelope (E), membrane (M), and nucleocapsid (N) proteins (Wang et al., 2020). The S protein facilitates viral entry into cells through the receptor angiotensin-converting enzyme 2 (ACE2) expressed in various cell types, including epithelial cells of the respiratory system (Salamanna et al., 2020). Hence, vaccinating against the S protein generates neutralizing antibodies and abrogates SARS-CoV-2 entry, making the S protein one of the most favorable targets in COVID-19 vaccine designs (Dai and Gao, 2021).

Oncolytic viruses (OVs) are a new class of therapeutics that can selectively infect and replicate in tumor cells, with the primary objective being the direct lysis of cancer cells. Infection with an OV results in a profound inflammatory reaction within the tumor, initiating innate and adaptive antitumor immune responses. Therefore, OV administration is a promising cancer treatment strategy (Kaufman et al., 2015). The recombinant vesicular stomatitis virus (VSV) vaccine platform has been widely used to combat several viral outbreaks, including those caused by Nipah (Foster et al., 2022; de Wit et al., 2023), Lassa (Safronetz et al., 2015; Cross et al., 2020), and Ebola (Henao-Restrepo et al., 2017) viruses. VSV∆51M is a VSV variant with deletion of methionine (M) 51 in the matrix gene. The deletion of this amino acid results in an impairment in the ability of the virus to block the antiviral interferon (IFN) response in normal tissues, increasing the safety profile of this variant (Stojdl et al., 2003; Wu et al., 2008). In our previous study (Alkayyal et al., 2023), we found that VSVΔ51M encoding the SARS-CoV-2 RBD had a significantly larger viral plaque surface area and higher viral titers than the parental virus, subsequently improving the viral spreading capacity. Moreover, we demonstrated that the presence of the SARS-CoV-2 RBD in the VSVΔ51M genome enhanced VSVΔ51M oncolytic activity *in vitro.* These findings suggest that our VSVΔ51M-RBD platform may have therapeutic potential as a vaccine for COVID-19.

Here, we report that utilizing the oncolytic VSV∆51M-RBD platform generated an anti-RBD humoral response *in vivo*, with a potentially good safety profile. Therefore, this vaccine warrants further preclinical and clinical investigation.

## Material and methods

### Construction of VSVΔ51M-RBD vaccine

The VSVΔ51M-RBDvirus was created as described previously (Alkayyal et al., 2023). Briefly, codon-optimized RBD gene, containing the amino acid region (319–541aa) of the full Spike gene of the SARS-CoV-2 (2019-nCoV), from the RBD expression plasmid (Sino Biological Inc., Beijing, China, cat# VG40592-UT) was inserted into a plasmid encoding the VSVΔ51M antigenome plasmid. The insertion was between the G and L genes, and primers used for this insertion as follows: Forward 5′-TGG​AAA​GTA​AGC​TAG​CTG​TAT​GAA​AAA​AAC​TCA​TCA​ACA​GCC​ATC​ATG​AGG​GTC​CAA​CCA-3′ and Reverse: 5′-GAA​GAA​TCT​GGC​TAG​CTC​AGA​AGT​TCA​CAC​ACT​TGT​TC-3’. These primers were designed to be compatible with the In-Fusion^®^ HD Cloning Kit (Takara Bio Inc., United States of America, cat# 638910). Both viruses were rescued in A549 cell line by infecting them with vaccinia virus expressing T7 polymerase and subsequently transfecting using Lipofectamine 2000 with 2 mg of VSV∆51M-RBD DNA plasmid together with plasmids encoding for VSV N, P and L (1, 1.25, 0.25 mg, respectively). The rescued virus was passaged and plaque purified, amplified and titrated on VERO cells.

**FIGURE 1:**
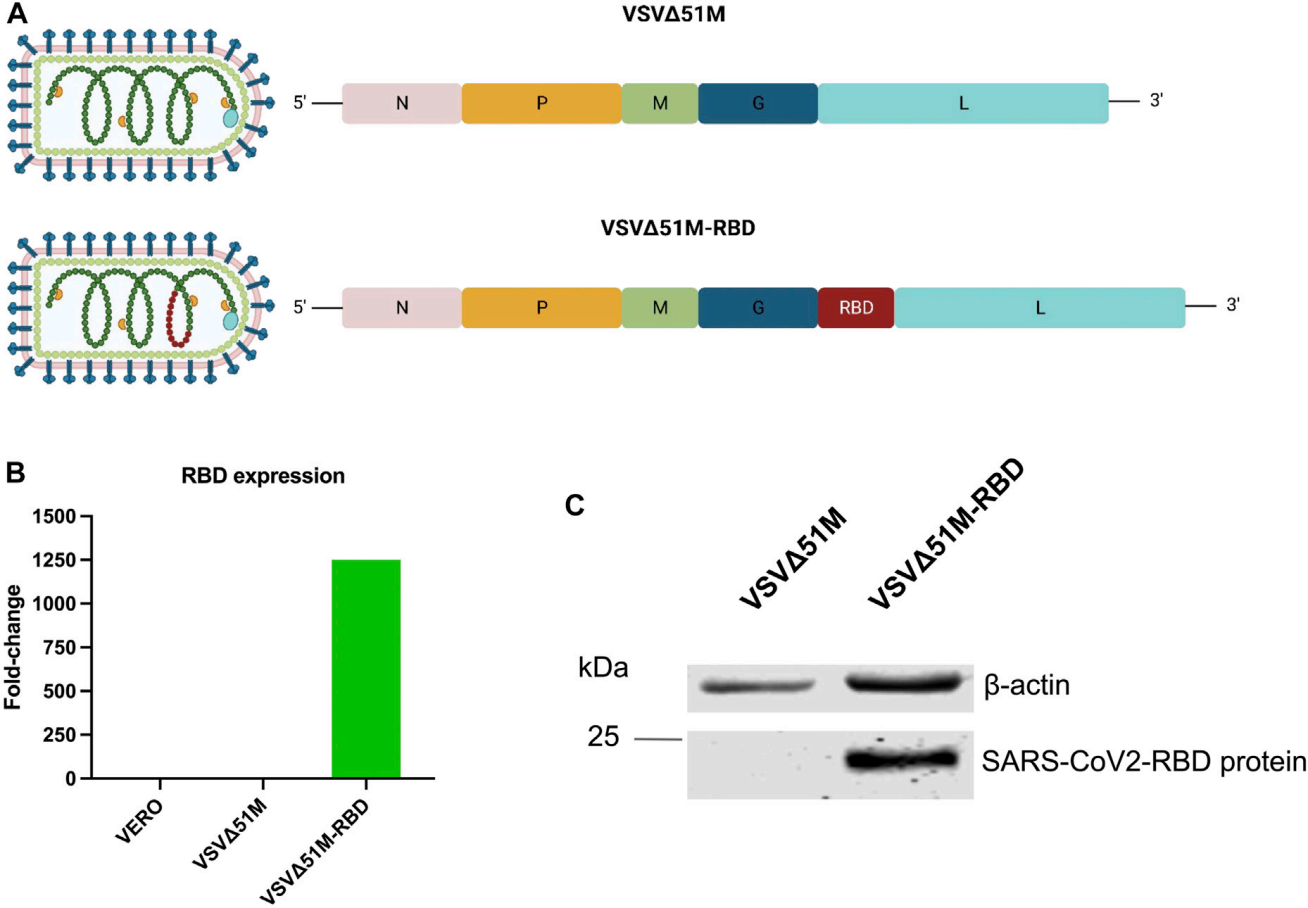
Generation of SARS-CoV-2 (2019-nCoV) Spike RBD VSV∆51M viral vaccine. **(A)** schematic diagram of the VSV∆51M-RBD viral genome highlighting the location of the SARS-CoV2 RBD insertion site. **(B)** RNA expression levels of RBD normalized to GAPDH in VERO - VSV∆51M-RBD–or VSV∆51M-infected cells, in comparison to uninfected VERO cells n = 1. **(C)** Protein expression levels of RBD in lysates of VSV∆51M-RBD infected VERO cells in comparison to VSV∆51M infected VERO cells. A band representing RBD ∼25 kDa was detected in VSV∆51M-RBD, while no band was observed in the VSV∆51M control. Figure 1A was created with BioRender.com under license agreement number NQ24WIY7X0.

### Cell lines and media

A549 human lung carcinoma and VERO cell lines were generous gifts from Mr. Suhail Melibary (King Abdulaziz University Hospital, Jeddah, Saudi Arabia). Both were cultured in Dulbecco’s Modified Eagle Medium (DMEM, Gibco) containing 10% fetal bovine serum (FBS), 2 mM L-glutamine, 1 mM sodium pyruvate, 100 units/mL penicillin and 100 μg/mL of streptomycin. Cells were maintained in humidified incubators at 37°C and 5% CO^2^ (Al-Musawi et al., 2020; Ibrahim et al., 2021).

### Propagation and purification of VSV∆51M and VSV∆51M-RBD viruses

VERO cells were infected with either VSV∆51M or VSV∆51M-RBD (MOI = 1). Post 24 h of infection, media was harvested with 20 mM EDTA, and centrifuged at 500 RCF for 10 min. The supernatant was filtered through a 0.22 µm pore size sterile filter and centrifuged at 21,000 RCF for 1 h and 30 min 4°C. Pellet was resuspended in 1x PBS. 20% sucrose was added to the virus and centrifuged at 41,000 RCF for 1 h and 30 min 4°C. Pellet was resuspended in 1x PBS. The virus was aliquoted and stored at −80°C.

### Virus titration and plaque assay

VERO cells were seeded in 12-well plates and infected by VSV∆51M-RBD 10x serial dilutions (10^2^—10^10^). After 1 h of incubation in a 37°C/5% CO^2^ incubator, agarose media (2:1 of 15% FBS DMEM media: 3% agarose) replaced the virus media. Post 24 h incubation at 37°C/5% CO^2^ incubator, media was removed and wells were covered by methanol—acetic acid fixative solution (3:1) for another 24 h at room temperature (RT). Agar was then removed and wells were rinsed with water. Wells were stained with Coomassie Blue (1mL/well) for 1 h RT. Finally, wells were rinsed with water and set to dry. Viral titers were calculated by means of the number of plaques in accordance with the viral dilution factor.

### Mice vaccination

For immune response studies, C57BL/6 or BALB/c female mice (6–10 weeks old) were vaccinated via the indicated route with the VSV∆51M-RBD vaccine or the parental vector for two doses, 2 weeks apart. The blood from individual mice was collected from the submandibular vein. After clotting of blood at room temperature, samples were centrifuged and serum was obtained. Serum samples were stored at 4°C until further analysis.

**For different routes study**, Immunizations were performed through; Intraperitoneal (IP), Intradermal (ID), Intravenous (IV), subcutaneous (SC), and intramuscular (IM) at 2 × 10^8^ PFU/mL. Two weeks after the boost immunization, blood samples were collected.

**For the dose escalation study**, Immunizations were performed intramuscularly on the hind leg. Mice were vaccinated once with either 5 × 10^7^, 1 × 10^8^, 5 × 10^8^ or 1 × 10^9^ PFU/mL. Two weeks after immunization, blood samples were collected.

### Western blot analysis

VERO cells were infected with VSV∆51M or VSV∆51M-RBD viruses at a multiplicity of infection (MOI) of 1. At 24 h post-infection, cells were collected and centrifuged at 500 *g* for 5 min. VSV∆51M- and VSV∆51M-RBD-infected cells were lysed in RIPA lysis buffer (Thermo Scientific) with protease inhibitor. Fifty µg of total proteins were loaded and separated on 8% SDS-PAGE. Proteins were transferred to nitrocellulose membranes, blocked with 5% skimmed milk for 1 h at room temperature then incubated with the primary antibody against SARS-CoV-2 RBD overnight at 4°C. Membranes were washed three times with 1x PBS-Tween for 5 min each and incubated with IRDye labeled secondary antibodies for 1 h at room temperature. After a series of washes (3 × 5 min), the protein bands were then visualized using an Odyssey Infrared Imaging System (LI-COR Biosciences). β-actin expression was used as an endogenous control for Western blotting. The primary antibodies used in Western blot experiments were as follows: SARS-CoV-2 (2019-nCoV) spike antibody, Mouse Mab (Sino Biological Inc., Beijing, China, cat# 40591-MM42, 1:1000), and monoclonal anti-β-actin (Abcam, United States of America 1:1000).

### RNA extraction for RT-qPCR analysis

VERO cells grown in a 12-well plate were infected with VSV∆51M or VSV∆51M-RBD, (MOI = 1) for 24 h in a 37°C/5% CO^2^ incubator. In each well, 500 µL of TRIzol Reagent (life technologies) were added to the cells. Samples were collected and stored at −80°C. On the day of RNA extraction, 200 μL of chloroform was added to each sample, incubated for 3 min, then centrifuged at 12,000 g for 10 min at 4°C. The clear aqueous top phase was recovered in a clean tube containing 500 μL of isopropanol. The tube was mixed by inversion and incubated at room temperature for 10 min. Samples were then centrifuged at 12,000 g for 10 min at 4°C, and the supernatant was discarded. The pellet was washed with 500 μL of 75% ethanol, vortexed, then centrifuged at 7,500 g for 5 min at 4°C and the supernatant was discarded. The pellet was dried at room temperature for 10 min and RNA was resuspended in 20 μL of RNase-free water and stored at—80°C until further analysis. The expression of the RBD was assessed using Quantifast SYBR Green RT-PCR (cat# 204156) relative to the GAPDH gene. The RBD gene was detected using the following primer pair: RBD Forward Primer (5′-GGA​GTG​AGC​CCA​ACC​AAA​CT-3′) and RBD Reverse Primer (5′-GGG​GCA​ATC​TGT​CTC​ACC​TC-3′). For the GAPDH gene, the primer pair used was: GAPDH Forward Primer (5′-TAA​ATT​GAG​CCC​GCA​GCC​TCC​C-3′) and GAPDH Reverse Primer (5′-GAC​CAA​ATC​CGT​TGA​CTC​CGA​CCT-3′). Negative controls were included using nuclease-free water. The reactions were performed on a QuantStudio 5 Real-Time PCR system (Applied Biosystems) (Al-Shammari et al., 2020).

### The detection of the RBD protein-specific IgG by indirect ELISA

SARS-CoV-2 spike RBD protein-specific IgG levels in mouse sera were determined by indirect ELISA. 96-well ELISA plates were coated with 1ug/mL SARS-Cov-2 Spike RBD recombinant protein (Sino Biological Inc., Beijing, China, cat# 40592-VNAH) overnight at 4°C. Plates were then washed with PBST, Phosphate Buffer Saline with 0.05% tween-20, (SIGMA) three times. Plates were then blocked with a BSA blocking buffer (PBST with 3% BSA (SIGMA)), for 2 h at room temperature (RT). Ten-fold serial diluted serum samples, starting with a 1:10 dilution, were incubated in the blocking buffer for 1 h at 37°C and then washed three times with PBST. Bound IgG was detected using HRP-conjugated goat anti-mouse IgG (at 1:2000) (EMD Millipore Corporation, Billerica, MA, United States of America cat# AP308P). Following a 1 h incubation at 37°C, washed plates were developed with 100 μL of the peroxidase substrate TMB (3,3′,5,5’ Tetramethylbenzidine) liquid substrate system for ELISA (SIGMA, United States of America, cat# 34021) for 20 min at RT. The reaction was quenched with 1N HCl (hydrochloric acid). Absorbance was read at 450 nm using an ELISA plate reader.

### Statistical analysis

All statistical analyses were performed using GraphPad Prism 9.0 software. Student’s t-test, one-way ANOVA with Tukey’s multiple comparisons test, and Dunnett’s multiple comparisons test were used as appropriate to determine statistical significance, with a cutoff of *p* = 0.05. The data are presented as mean ± SD (Bahjat et al., 2021).

## Results

### Development of the VSV∆51M-RBD vaccine

To generate a VSV∆51M-RBD vaccine candidate, the RBD coding sequence of SARS-CoV-2 was inserted into the VSV∆51M genome between the glycoprotein (G) and polymerase (L) genes (Figure 1A). To confirm RBD expression by the rescued VSV∆51M-RBD vaccine, we infected VERO cells with either VSV∆51M or VSV-Δ5M1-RBD and evaluated the expression level of the RBD gene in the infected cells by using qRT‒PCR. Indeed, the expression level of the RBD gene produced by VSV-Δ5M1-RBD (in terms of the RBD gene copy number) exhibited an approximately 1250-fold increase, but this was not the case for uninfected VERO cells or VSVΔ51M infected cells, in which the RBD gene signal was not detectable (Figure 1B). To further evaluate the expression of the RBD at the protein level, we performed a Western blot analysis of VERO cells infected with either VSVΔ51M or VSV-Δ5M1-RBD. As expected, we were able to detect RBD protein expression in the VSV-Δ5M1-RBD-infected cells, with a band at ∼25 kDa corresponding to the size of the inserted RBD gene, suggesting that the generated vaccine could infect and express the RBD protein (Figure 1C).

### The VSV∆51M-RBD virus retains its cytotoxicity and viral fitness

**FIGURE 2:**
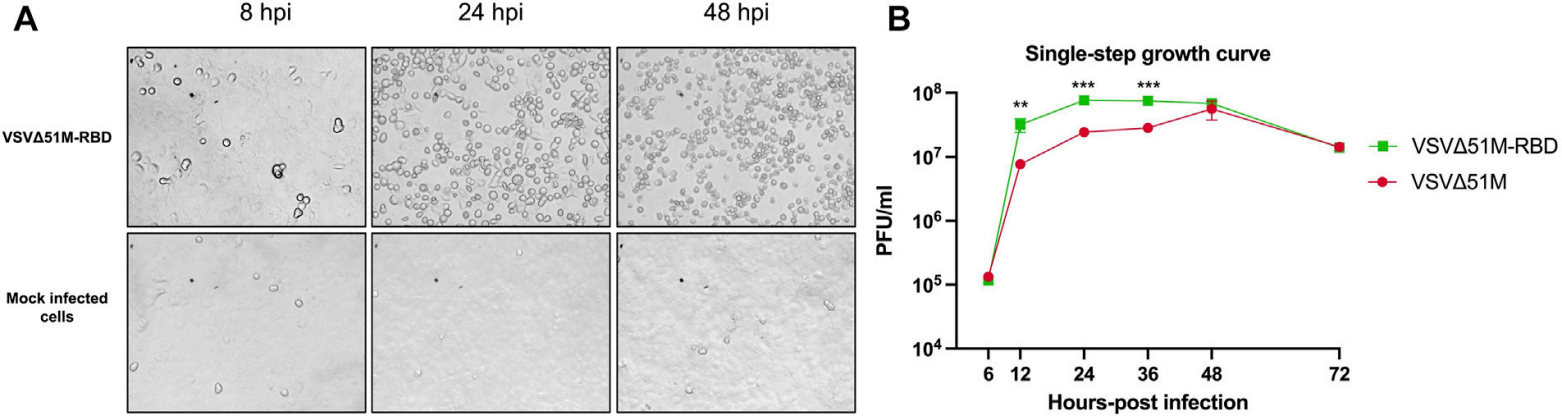
Characterization of VSV∆51M-RBD. VERO cells were infected with VSV∆51M-RBD at an (MOI = 1). **(A)** Images were captured at 8-, 24- and 48-h post-infection (hpi) using a phase-contrast microscope (data are represented at ×20 magnification). **(B)** Single-step growth kinetics of VSV∆51M and VSV∆51M-RBD. VERO cells were infected with the recombinant VSV viruses (MOI = 3) and virus titers were measured at the indicated time points post-infection by plaque assay. *p* < 0.05, **p < 0.005, ***p < 0.0005 by *t*-test.

To confirm the retained cytotoxicity of VSV∆51M-RBD, we infected a VERO cell monolayer with VSV∆51M-RBD at a multiplicity of infection (MOI) of 1 and evaluated the cytopathic effect (CPE) of the virus at different time points. Images of VSV∆51M-RBD-infected VERO cells acquired at 8, 24 and 48 h post-infection (hpi) indicated that the CPE increased with time, suggesting that the cytotoxicity of the VSV∆51M-RBD virus was retained (Figure 2A). To determine if the insertion of the SARS-CoV-2 RBD gene into the VSV∆51M genome attenuated viral cytotoxic activity, a single-step growth curve was generated by infecting VERO cells with either VSV∆51M or VSV∆51M-RBD and evaluating viral production at 6, 12, 24, 36, 48, and 72 h post-infection. As expected, the production rates of both viruses increased gradually from 6 to 12 h and plateaued at 24 h (Figure 2B). Interestingly, VSV∆51M-RBD resulted in significantly higher viral titers at 12, 24 and 36 h post-infection (*p* < 0.05, **p < 0.005, and ***p < 0.0005, respectively). Collectively, these observations suggest that the insertion of the SARS-CoV-2 RBD gene into the VSV∆51M genome did not attenuate VSV∆51M viral cytotoxicity or fitness.

### The VSV∆51M-RBD vaccine is safe and immunogenic

**FIGURE 3:**
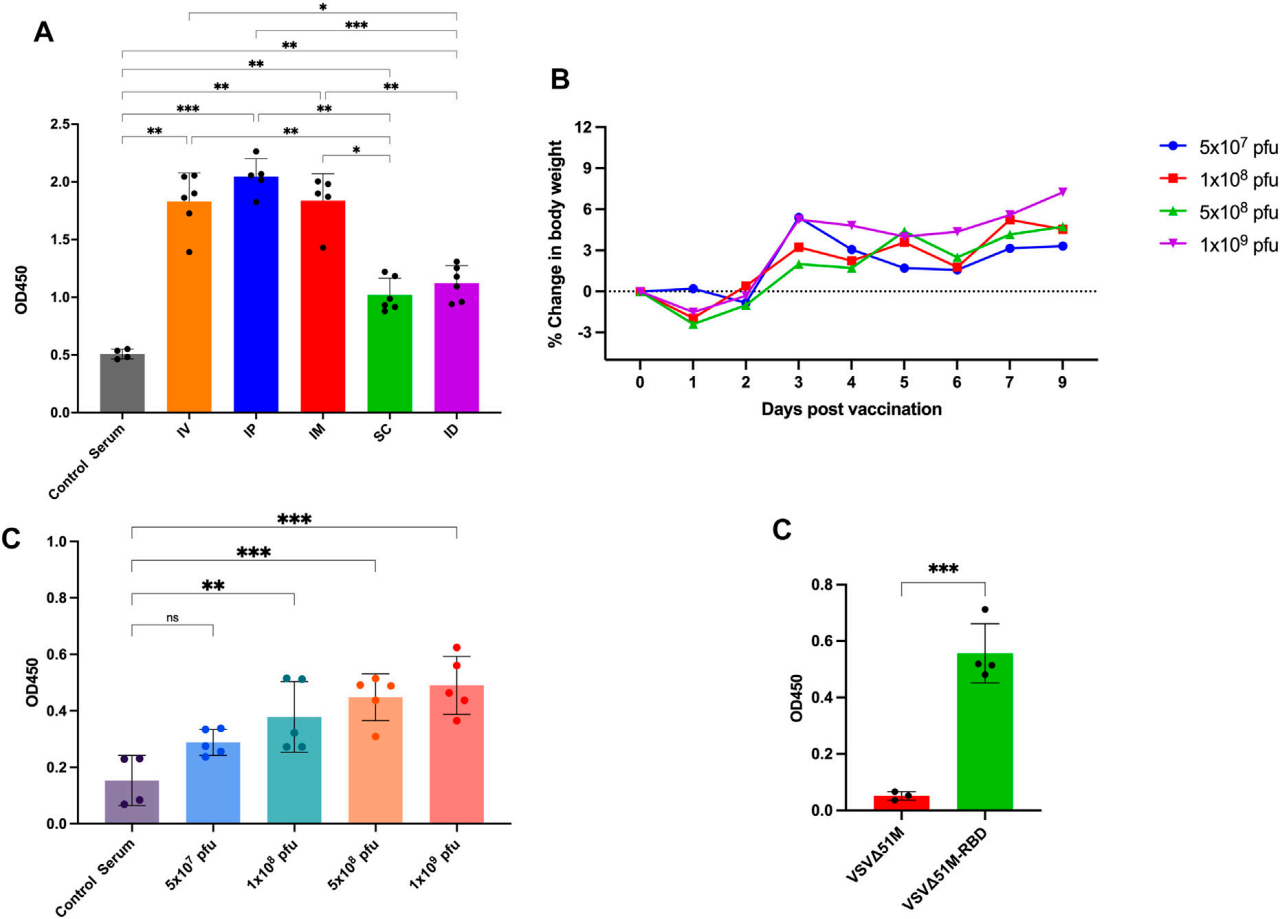
Immunogenicity of VSV∆51M-RBD in mice. **(A)** Humoral immune responses were assessed by RBD-specific ELISA for administering the vaccine through different routes; IP, IM, IV, ID, SQ in C57BL/6 mice at week 2 post-immunization **(B)** Average body weight of vaccinated mice with different doses; 5 × 10^7^, 1 × 10^8^, 5 × 10^8^ or 1 × 10^9^ PFU/mL, in a dose escalation manner. After vaccination, mice were weighed daily. Percent body weight per group was calculated compared to body weight at the time of vaccination. **(C)** The corresponding humoral immune responses for different doses; 5 × 10^7^, 1 × 10^8^, 5 × 10^8^ or 1 × 10^9^ PFU/mL at week 2 post-immunization **(D)**. The detection of RBD antibodies in VSV∆51M-RBD vaccinated Balb/c mice. Serum was collected from all animals 2 weeks post booster immunization. IgG titers were determined by ELISA against the recombinant VSV∆51MRBD. N = three to five biologically independent animals per group. Error bars indicate as ± SD. *p* < 0.05, **p < 0.005, ***p < 0.0005, by one-way ANOVA with Tukey’s multiple comparisons test for panel A, Dunnett’s multiple comparisons test for panel C, and a Student’s t-test for panel D.

To evaluate the immunogenicity of our COVID-19 vaccine candidate, we first wanted to determine the best route of vaccination for inducing an anti-RBD response. We vaccinated naive C57BL/6 mice with the VSV∆51M-RBD vaccine twice (a priming dose on Day −14 and a booster dose on Day 0) using five different routes (IP, intraperitoneal; ID, intradermal; IV, intravenous; SC, subcutaneous; and IM, intramuscular) (Figure 3A). Two weeks after the booster vaccination, we found that the serum anti-RBD level was detectable for all vaccinated routes but to a greater extent when mice were vaccinated via the IP, IM, or IV route compared to the SC and ID routes, and these increases were statistically significant (IP vs. SC, IP vs. ID, IP vs. Control Serum, IM vs. SC, IM vs. ID, IM vs. Control Serum, SC vs. IV, SC vs. Control Serum, ID vs. IV, ID vs. Control Serum, IV vs. Control Serum. This observation suggests that the VSV∆51M-RBD vaccine is immunogenic. Hence, we selected the IM route for subsequent experiments due to its ease of testing in the clinical development stage.

Next, we performed a dose escalation experiment to evaluate whether the VSV∆51M-RBD vaccine could be tolerated in doses that have been reported to be tolerable in mice (Kim et al., 2022). Based on our observation of the anti-RBD response induced by IM vaccination with VSV∆51M-RBD, we vaccinated naive mice with a single dose of VSV∆51M-RBD administered intramuscularly at different doses (5 × 10^7^, 1 × 10^8^, 5 × 10^8^ and 1 × 10^9^ PFU). As expected, the mice vaccinated with 1 × 10^8^, 5 × 10^8^ or 1 × 10^9^ PFU experienced flu-like symptoms, and transient weight loss was observed 24 hours after vaccine administration but rebounded beginning at 48 hours (Figure 3B). Concomitantly, the levels of anti-RBD antibodies in the serum of the vaccinated mice were assessed at week two after vaccination and were correlated with the vaccination dose (Figure 3C). The levels of anti-RBD antibodies induced by the 1 × 10^8^, 5 × 10^8^ and 1 × 10^9^ PFU doses were significantly higher compared to serum from unvaccinated. Furthermore, we wanted to assess whether this immunogenicity could be replicated in another mouse strain. Therefore, we vaccinated BALB/C mice with 2 × 10^8^ PFU/mL VSV∆51M or VSV∆51M-RBD administered IM. As shown in Figure 3E, this vaccination approach resulted in a significantly higher serum anti-RBD level than the injection of the VSV∆51M virus at 2 weeks after the booster vaccination (*p*-value <0.005). Taken together, these results provide supportive evidence that the VSV∆51M-RBD vaccine is safe and immunogenic *in vivo* when tested in mice.

## Discussion

The goal of this study was to repurpose the oncolytic virus VSV∆51M for COVID-19 vaccination. Here, we generated a VSV∆51M-RBD vaccine candidate by inserting the RBD coding sequence of SARS-CoV-2 into the VSV∆51M genome. Then, by comparing VSV∆51M-RBD to the parental VSV∆51M virus, we determined that the SARS-CoV-2 RBD gene did not negatively attenuate VSV∆51M viral cytotoxicity or fitness. Additionally, we demonstrated that the VSV∆51M-RBD vaccine was safe and immunogenic *in vivo*.

The VSV∆51M-RBD vaccine has unique advantageous characteristics. The VSV vaccine platform can generate a rapid and effective immune response, and it is suitable for efficient large-scale virus production (Patel et al., 2015; Fathi et al., 2019). Therefore, it has been clinically used and preclinically investigated to combat several viral outbreaks, including the SARS-CoV-2 pandemic (Dieterle et al., 2020; Yahalom-Ronen et al., 2020; Kim et al., 2022). In this study, we employed an attenuated VSV strain, VSV∆51M, which harbors the deletion of methionine (M) 51. This genetic alteration makes the VSV∆51M variant more sensitive to the interferon (IFN) response (Stojdl et al., 2003; Wu et al., 2008). Hence, VSV∆51M not only retains the advantages of VSV but is also much safer than wild-type VSV. Hence, VSV∆51M not only retains the advantages of VSV but is also much safer than wild-type VSV. Additionally, the insertion of the SARS-CoV-2 RBD gene into the VSV∆51M genome significantly improved the viral replication capacity (Alkayyal et al., 2023). This novel observation has been employed when utilizing VSV∆51M-RBD as an anticancer agent with a better therapeutic index than the parental VSV∆51M virus owing to its enhanced replication and spreading in cancer cells. Moreover, previous studies have shown that the spike protein of SARS-CoV-2, particularly the S1 region containing the receptor-binding domain (RBD), can interfere with and downregulate the production and signaling pathway of type I interferon (IFN-I) by suppressing the phosphorylation and nuclear translocation of STAT1 and disrupt its interaction with JAK1 (Zhang et al., 2021). However, in our study, we found that the insertion and expression of the RBD antigen in the VSV∆51M platform did not negatively impact viral replication probably owing to its interstice interferon sensitivity. The VSV∆51M-RBD virus retained its cytotoxicity and viral fitness, indicating that the presence of the RBD gene did not attenuate the virus.

VSV∆51M-RBD is potentially an effective booster vector for adenovirus-based COVID-19 vaccines. There are currently four different adenovirus-based COVID-19 vaccines approved for emergency use. These vaccines are Janssen Ad26.COV2.S, Oxford/AstraZeneca ChAdOx1 nCoV-19 (AZD1222), Sputnik V Gam-COVID-Vac and Convidecia Ad5-nCoV. However, adenovirus-based vaccines typically require a booster immunization to enhance the duration and potency of the adenovirus-elicited immune responses (Bridle et al., 2013). In alignment with our strategy for developing rhabdovirus-based COVID-19 vaccines, there is compelling evidence in the literature suggesting that rhabdoviruses, including VSV (Bridle et al., 2013) and Maraba virus (Pol et al., 2019), can further improve the quantity and quality of T cells when used as booster vaccines with adenovirus-based priming vectors. Given that the VSV∆51M-RBD vaccine is a rhabdovirus, it is likely that heterologous immunization mediated by boosting with our VSV∆51M-RBD viral vector after priming with an adenovirus-based vaccine could be an efficient booster strategy for COVID-19 vaccination. The heterologous prime-boost approach has been investigated in the context of COVID-19 vaccination before by utilizing an adenovirus-based vaccine (ChAdOx1 nCoV-19) priming immunization with an mRNA (BNT162b2) booster vaccination, which revealed that this vaccination strategy elicits potent humoral and cellular immunity against prevalent SARS-CoV-2 variants (Gross et al., 2022). Given the limited access to the biosafety level 3 (BSL-3) containment facilities required to handle SARS-CoV-2 safely, we were not able to conduct efficacy studies for the VSV∆51M-RBD vaccine. Nonetheless, the use of the VSV∆51M-RBD vaccine did not lead to serious side effects and was able to elicit a humoral immune response, which might provide protective immunity against SARS-CoV-2. These findings warrant further investigation and potential translation into the clinical trial setting.

## Data Availability

The raw data supporting the conclusion of this article will be made available by the authors, without undue reservation.
